# Modified Lignin-Based Cement Solidifying Material for Improving Engineering Residual Soil

**DOI:** 10.3390/ma16227100

**Published:** 2023-11-09

**Authors:** Xiang Yu, Hongbo Lu, Jie Peng, Jinming Ren, Yongmin Wang, Junhao Chen

**Affiliations:** 1 Power China Huadong Engineering Corporation Limited, Hangzhou 311122, China; yu-xiang@hhu.edu.cn (X.Y.); ren_jm@hdec.com (J.R.); wang_ym2@hdec.com (Y.W.); chen_jh4@hdec.com (J.C.); 2 College of Civil and Transportation Engineering, Hohai University, Nanjing 210098, China; peng-jie@hhu.edu.cn

**Keywords:** solidifying agent, lignin, hydroxylation, solidification mechanism

## Abstract

Although lignin improves the strength and modulus of soil, it is less active when unmodified, and it exhibits more limited effects on soils in combination with traditional Ca-based curing agents. Lignin-solidified soil also exhibits deficiencies, such as poor durability under dry–wet cycling conditions, and thus, the amelioration effect is limited. This study investigated the enhancement of cement-solidified soil using hydroxylated lignin with sodium silicate and quicklime used as activators to improve the engineering performance and durability of the treated soil. Using respective cement, sodium silicate, quicklime, and lignin contents of 7%, 0.4%, 0.2%, and 0.2% with respect to the dry mass of the slag soil, the strength and cohesion of the composite-solidified soil were 1.5 times those of cement-solidified soil, whereas the internal friction angle increased by 5.1°. At a solidifying age of 14 d, the penetration resistance almost doubled, indicating a significant improvement in the bearing capacity of the soil. The results suggest that modified lignin-based admixtures may significantly enhance the performance of cement-solidified soil. The cement curing admixture used in this study provides theoretical and technological support for curing agent preparation and the utilization of slag.

## 1. Introduction

As the development of infrastructure construction progresses, activities such as the development of underground spaces and dredging projects result in a significant amount of construction slag characterized by a large porosity ratio and high compressibility and sensitivity, posing engineering challenges [1,2,3]. Currently, the primary method of managing construction slag involves external transportation and landfilling, which is not only costly but also results in substantial resource waste. Consequently, in the context of the current global emphasis on sustainable development, identifying a reasonable method of disposing of these excavation slags has emerged as an urgent engineering and environmental issue.

Slag soil curing technology, which is used as an economical, simple curing technology to form the corresponding curing products, is one of the optimal methods of addressing the formation of construction slag. The curing agent is critical in slag soil curing and solidifying agents, which are significant auxiliary materials in the construction industry and are extensively utilized in soil solidification, enhancing the bearing capacity of soil and reducing construction costs. Traditional cement solidifying agents enhance the strength of construction slag [4,5,6], but their production processes pose environmental pollution issues [7], and thus, researchers are exploring green, energy-efficient, economical, and effective soil improvers. Recent studies have investigated the use of various industrial by-products to enhance soil, aiming to realize a “waste-treating-waste” effect [8,9,10]. Yadu [11] employed granulated blast furnace slag in soil solidification. Unconfined compressive strength (UCS) studies revealed that soil solidified with 9% granulated blast furnace slag was approximately 28% stronger than plain soil, but the excessive addition of blast furnace slag inhibited soil strengthening. Horpibulsuk et al. [12] improved Thai silty clay using carbide slag and fly ash, and the strength of the solidified soil increased with the carbide slag content. Ning et al. [13] used cement, fly ash, and coal slag in sludge solidification, and mixing these three materials could solidify heavy metals and organic components within the sludge, thereby reducing the amounts of harmful components in its leachate to satisfy the national standards. Although such industrial by-product solidifying agents may relatively solidify soil, large dosages are generally required, and their utilization is often regionally restricted, which limits their application. Therefore, studying the use of minimal materials combined with traditional solidifying agents to enhance the performance of construction slag is crucial in the utilization of slag.

Lignin, which is an organic polymer widely distributed in nature and a low-cost by-product of the paper industry, enhances the soil strength and modulus and may be used to overcome several of these limitations, thus exhibiting considerable application prospects [14,15,16,17]. Ceylan et al. [18] treated subgrade cohesive soil with two different types of industrial by-product lignin, and lignin A was more effective in improving the soil strength under low-water-content conditions, whereas lignin B improved the soil strength under higher-water-content conditions. Indraratna et al. [19] reported that lignosulfonate and cement could increase the resistance of fine sand toward water erosion, and Tingle et al. [20,21] improved clay and silty sand using seven different improvers, such as lignin, enzymes, and resins. Compared to those of the other six materials, lignin significantly improved the soil strength, and the water stability of the improved soil was superior. Despite these qualities, unmodified lignin exhibits low activity and limitations such as the poor durability of the solidified soil under conditions including variations in temperature [21,22,23]. Therefore, modifying lignin is necessary to enhance the durabilities and mechanical properties of lignin-based materials for more efficient application in soil solidification.

Numerous methods of modifying lignin are reported, with the prevalent modification techniques encompassing nitrification, etherification, esterification, and hydroxylation [24]. Hydroxylation of lignin, in particular, may regulate its molecular weight and augment the hydroxyl content, thereby enhancing its reactivity [25]. However, little research regarding the use of modified lignin materials as curing additives in soil stabilization has been conducted. Therefore, this study investigates the enhancement of cement-solidified soil using modified lignin-based solidifying materials. Minimal amounts of sodium silicate and quicklime are used to modify and activate industrial by-product lignin to yield hydroxylated lignin materials. This approach enables the coordinated utilization of slag soil with traditional cement solidifying agents, reduces the dosages and costs of the solidifying agents, and facilitates the efficient use of industrial by-product lignin in the field of solidified soil. Additionally, characterization methods, such as X-ray diffraction (XRD), scanning electron microscopy (SEM), and Fourier transform infrared (FTIR) spectroscopy, are utilized in analyzing the mechanisms of action of the solidifying materials. The results suggest that modified lignin-based admixtures may significantly enhance the performance of cement-solidified soil. This study provides theoretical and technical support for the development of lignin modification and solidified soil technology and promotes the application of solidified soil technology in roadbed construction.

## 2. Materials and Methods

### 2.1. Test Materials

**Figure 1:**
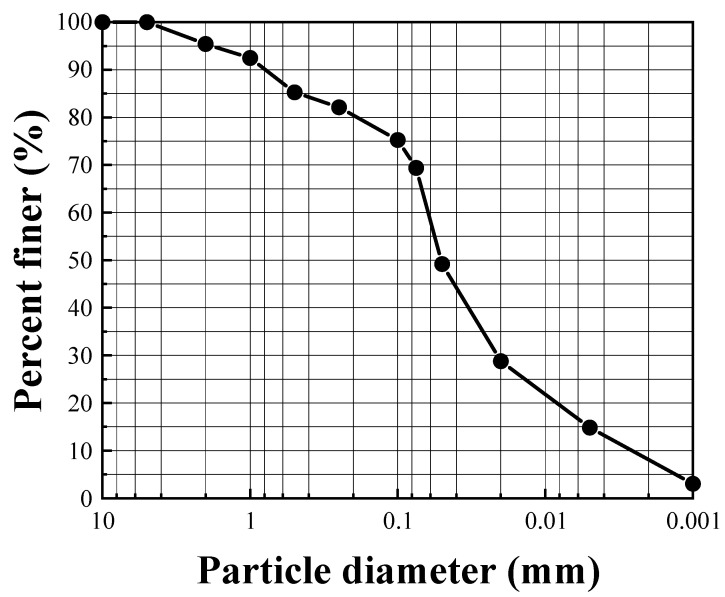
Slag grading curve of the soil.

The soil used in this study originated from the construction site of a wind farm road construction project in Yangzhou City. The particle size distribution of the test soil was evaluated using a screening method and Mastersizer 3000 laser particle size analyzer (Malvern Panalytical, Malvern, UK). The obtained grading curve, as shown in Figure 1, represents low-liquid-limit mucky clay. Its basic physical properties and main chemical composition are shown in Table 1 and Table 2, respectively. The solidifying agent employed was P·O 42.5 ordinary Portland cement, and its composition is shown in Table 2. The solidifying materials comprised lignin, sodium silicate, and quicklime. The industrial-grade lignin used was a yellow-brown powder with a slight fragrance, and it was sourced from China Shandong Tianfeng Chemical Technology. The sodium silicate, which was an industrial-grade product of You Rui, was a colorless and slightly colored transparent viscous liquid. The quicklime was an industrial-grade powder produced by Tengshun Calcium Industry, Yichun, China; the chemical composition of the quicklime is shown in Table 3, and the characteristics of sodium silicate are shown in Table 4.

### 2.2. Solidified Soil Performance Study

#### 2.2.1. UCS Study

First, the soil samples collected from the site were dried in an oven and then crushed. The soil was passed through a standard sieve with a pore size of 2 mm, and the dry soil was evaluated after screening. The mold utilized in preparing the UCS specimens measured 8 and 3.91 cm in height and diameter, respectively. Prior to specimen preparation, the water content of the soil sample was adjusted to 20% (approximating the water content in working conditions), and varying proportions of solidifying agents were added in three layers for compaction. Sodium silicate and lignin were dispersed in water and incorporated into the soil via stirring, whereas the quicklime powder was directly mixed into the soil (the mixing ratios were calculated based on the dry mass of the slag soil and maintained in subsequent experiments; material sizes and mixing methods consistent with these were used in subsequent studies). The specimens prepared using different proportions were subsequently placed in a standard solidifying room (temperature = 20 ± 2 °C, relative humidity ≥ 95%) to solidify until the corresponding age, at which point their compressive strengths were measured using a fully automatic unpressurized compressive strength tester produced by China Beijing Huakan Science and Technology. The UCS study was performed using a strain-controlled application of the test load at a strain rate of 2%/min. The influences of the contents of the different materials on the UCS of the solidified soil were explored by studying the influences of admixtures with different contents on the physical and mechanical properties of the test soil. Considering the previous research and the situation of the project, the UCS of the 7 d curing age was employed as the test index, and the contents of cement, sodium silicate, quicklime, and lignin were controlled to produce cement-, cement-lime-, cement-sodium silicate-, cement-lignin-, and composite-solidified soil samples. The single-doped and orthogonal test schemes of the cement-based materials used are summarized in Table 5 and Table 6.

#### 2.2.2. Direct Shear Study

The preparation of direct shear specimens involved the use of a ring knife sampler to compress the soil samples with varying proportions of solidifying agents (Table 7) into rings with respective inner diameters and heights of 61.8 and 20 mm. Following preparation, the specimens were placed in a standard solidifying room until they reached the corresponding age, at which point their shear strengths were measured using a ZJ strain-controlled direct shear instrument manufactured by China Nanjing Ningxi Soil Instrument. During the study, the vertical pressure was controlled at 100, 200, 300, or 400 kPa, and the shear rate was 1.2 mm/min. The dynamometer readings were recorded during the study, and when they no longer increased and retreated rapidly, the sample was sheared. After shearing was completed, the handwheel was reversed, the vertical pressure, frame, pressure cover, and other devices were rapidly removed, and the sample was then removed.

#### 2.2.3. Dry–Wet Cycling Study

An anti-dry–wet cycling performance study was conducted using soil samples improved with the different solidifying agents (Table 7), referencing the American material test standard ASTM D4843-88 “Standard Test Method for Wetting and Drying Test of Solid Wastes”. The drying temperature was set at 45 °C to avoid excessively high temperatures that could accelerate the hydration reaction [26]. The soil samples cured with different modifiers were subjected to ten dry–wet cycles, and each dry–wet cycle lasted 48 h. During drying, the sample was heated in an oven at 45 °C for 24 h, whereas during wetting, the sample was soaked in distilled water at 20 °C for 24 h. After each cycle, the sample was rapidly wiped with filter paper to remove water from the surface, the sample was weighed, and its mass loss was recorded. The UCS of the sample was recorded after the third, fifth, and tenth cycles.

#### 2.2.4. Dry Shrinkage Study

Soil samples enhanced with the various solidifying agents (Table 7) were converted via static pressure to rectangular specimens measuring 50 × 50 × 200 mm^3^, using a small-beam dry shrinkage sample mold. These specimens were then placed in a standard solidifying room for a solidifying period of 7 d. Following solidification, the specimens were saturated with water and then placed in a dry shrinkage room at a respective temperature and relative humidity of 20 °C and 60% for evaluation using a small-beam shrinkage tester. The mold and equipment used in evaluating the shrinkage performances of the cured soils were produced by Anruida Instrument Equipment (Cangzhou, China).

#### 2.2.5. Lightweight Dynamic Cone Penetration (DCP) Study

Lightweight DCP is a method that utilizes the kinetic energy of a constant-mass hammer to drive a cone probe into the soil layer. The engineering properties of the soil body are then assessed based on the ease of probe penetration [27]. The levels of effectiveness of solidifying agents and cement in improving engineering slag soil are evaluated based on two indicators: the DCP index (DCPI) and penetration resistance (*Rs*). The DCPI represents the penetration depth of each probe into the soil body, whereas *Rs* indicates the resistance encountered by the probe per unit depth during penetration.

The DCP study was employed to assess the on-site strengths of engineering slag before and after enhancement using external solidifying materials. The penetration was recorded at intervals of five hits, with the DCPI and *Rs* values reflecting the results [27]. The DCP indicators were evaluated using a DCP instrument produced by Hebei Star Blue Building Instrument (Cangzhou, China). The effects of 7% cement and composite curing agents were compared and analyzed.

### 2.3. Solid Sample Characterization

The chemical compositions of the cement and soil samples were determined using X-ray fluorescence spectrometry (Axios X, Malvern Panalytical, Malvern, UK), and SEM (Regulus8100, Hitachi, Tokyo, Japan) was used to analyze the microscopic morphological changes in the soil samples before and after solidification. First, trace samples of vegetal and various modified soils were removed from the specimens after drying, grinding, and sieving and directly adhered to a conductive adhesive. The samples were then sprayed with Au for 45 s using an SC7620 sputter coater (Quorum Technologies, Laughton, UK) at 10 mA, and then their morphologies were observed at different magnifications using SEM. The phase structures of the soil samples pre- and post-solidification were characterized using XRD (Ultimate IV, Rigaku, Tokyo, Japan). After 7 d of maintenance, the untreated and cured soil specimens with different amendments were dried and ground into powders with a mortar and pestle and then evaluated using XRD in the scanning angle range 5°–90° at a scanning speed of 5°/min. FTIR spectroscopy (Nicolet iS20, Thermo Fisher Scientific, Waltham, MA, USA) was employed to evaluate the changes in the functional groups of the soil samples before and after solidification. After drying, grinding, and sieving the slag soil treated using the cement, cement–lime, cement–sodium silicate, or composite curing agent for 7 d, a small amount of the sample and an appropriate amount of dry KBr powder were placed in a mortar in a dry environment. The powder mixture was ground several times until completely ground and then placed in a tablet press to press the tablet for use in FTIR spectroscopy. The background and infrared spectra of the samples were collected using a respective resolution, scan number, and wavenumber range of 4 cm^−1^, 32, and 400–4000 cm^−1^.

## 3. Results and Discussion

### 3.1. UCS

#### 3.1.1. Single-Dopant Study

**Figure 2:**
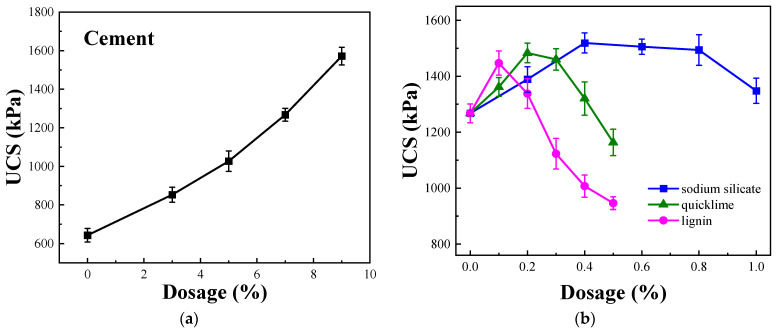
Unconfined compressive strength at different material contents. (**a**) Different cement dosages and (**b**) 7% cement mixed with different materials.

Figure 2a shows the results of the UCS evaluation of soil doped with cement. Clearly, the UCS of the solidified soil increases incrementally with increasing cement content. The strength reaches 1267 kPa when the cement content is 7%, which is almost double the strength of plain soil. However, excessive cement addition may lead to dry shrinkage of the solidified soil and reduced durability under dry–wet cycling [28,29]. Therefore, a cement content of 7% is used as the benchmark ratio in subsequent research regarding composite solidifying agents.

Sodium silicate, quicklime, and lignin were separately added at a cement benchmark ratio of 7%. Figure 2b shows that the UCS of the solidified soil initially increases and then decreases with increasing sodium silicate content. A noticeable inflection point occurs close to a sodium silicate content of 0.4%. At lower sodium silicate contents, the strength rapidly increases with increasing sodium silicate content, reaching a maximum of 1518 kPa, which is 20% higher than the strength of 7% cement-solidified soil. An optimal dosage is observed close to a quicklime content of 0.2%, where the strength is 1484 kPa, which is 17% higher than that of cement-solidified soil. Similarly, after adding lignin, the UCS of cement-solidified soil initially increases and then declines as the lignin content increases. A clear inflection point is observed when the lignin content is 0.1%, where the strength of the solidified soil is 1446 kPa, which is 14% higher than that of cement-solidified soil. Therefore, unmodified lignin may exhibit a low activity and induce a limited increase in the strength of cement-solidified soil. Similar results were obtained by Santonia et al. [30], who used unmodified lignin in conjunction with cement in soil improvement, indicating that lignin does not effectively promote cement hydration.

#### 3.1.2. Orthogonal Study

Based on the results, an orthogonal study was conducted using a cement benchmark ratio of 7%. Sodium silicate contents of 0.2%, 0.4%, and 0.6%; quicklime contents of 0.1%, 0.2%, and 0.3%; and lignin contents of 0.1%, 0.15%, and 0.2% were employed.

The results of the orthogonal study comprising nine groups indicate that the UCSs of the specimens improve to varying degrees compared to that of cement-solidified soil under different proportions of external materials (Table 8). The optimal group comprises 0.4% sodium silicate, 0.2% quicklime, and 0.2% lignin. The UCS may reach 1925 kPa, which is 52%, 30%, 27%, and 33% higher than the maximum UCSs of cement-solidified soil and cement-solidified soil doped with lime, sodium silicate, or lignin (1267, 1484, 1518, and 1446 kPa), respectively. This suggests that the modified lignin-based material significantly enhances the strength of cement-solidified soil.

### 3.2. Direct Shear Study

**Figure 3:**
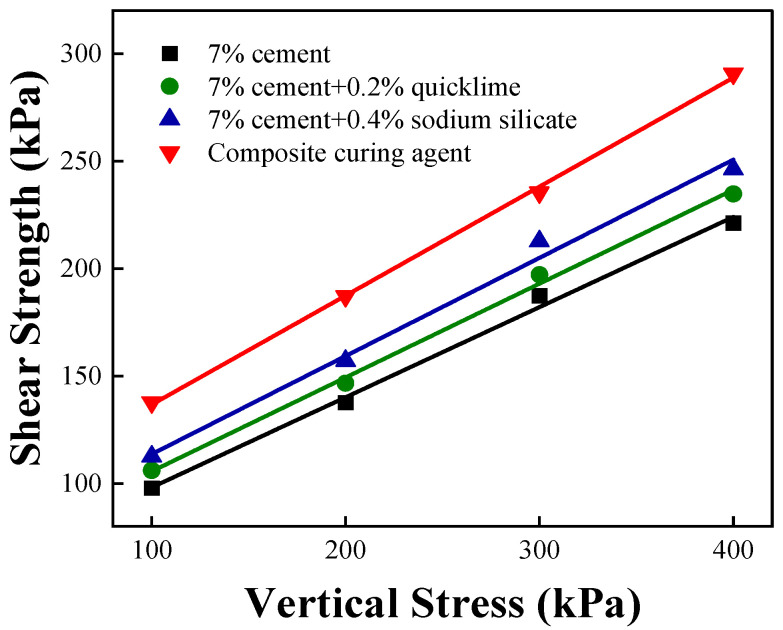
Relationship between shear strength and vertical stress.

Figure 3 shows the relationships between the vertical stresses and shear strengths of four improved soil samples after a solidifying period of 7 d. The shear strength increases incrementally with increasing vertical stress. Strong linear relationships between the vertical stresses and shear strengths of the four solidified soils are observed. The cohesion and internal friction angle, which are significant indicators of the mechanical properties of soil, are also considered, and the shear strength parameters of the four solidified soils are shown in Table 9.

Various materials enhance the cohesion and internal friction angle of cement-solidified soil (Table 9), but the inclusion of quicklime results in mere respective increases of 5.9 kPa and 1.0° in the cohesion and internal friction angle. The cohesion and internal friction angle of cement-solidified soil are, respectively, increased by 11.7 kPa and 2.2° with the addition of sodium silicate. The most significant improvement in the shear strength index of cement-solidified soil is observed upon the addition of the modified lignin-based material. The cohesion and internal friction angle, respectively, increase from 56.2 to 86.0 kPa and 24.0° to 29.1°, marking respective increases of 29.8 kPa and 5.1°. Notably, the cohesion is 1.5 times that of cement-solidified soil. The increases in the cohesion and internal friction angle of the soil body are larger after treatment with modified lignin-based materials compared to those after treatment with inorganic-binder solidifying agents. Therefore, modified lignin-based materials enhance the shear strength of cement-solidified soil.

### 3.3. Dry–Wet Cycling Studies

**Figure 4:**
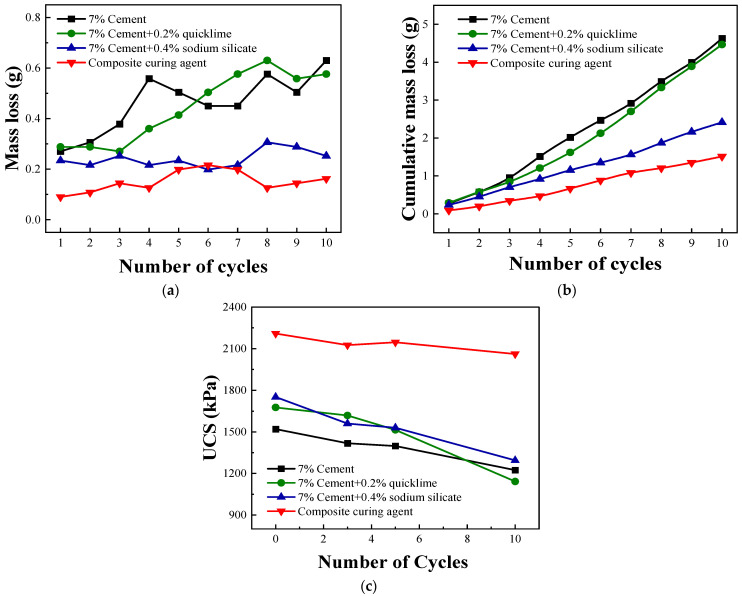
Relationships between the dry–wet cycling indices and number of cycles. (**a**) Cyclic and (**b**) cumulative mass losses and (**c**) unconfined compressive strength.

Dry–wet cycling studies of the cement-, cement–lime-, cement–sodium silicate-, and composite-solidified soils provide insights into their durability performances under dry–wet cycling. The mass losses of cement- and cement–lime-solidified soils increase gradually with the number of cycles from initial cycle losses of 0.3 g to approximately 0.6 g (Figure 4a). In contrast, the mass losses of cement–sodium silicate- and composite-solidified soils remain relatively stable, fluctuating by 0.2 g per cycle and remaining between 0.1 and 0.2 g per cycle, respectively. These values are lower than the previous three. The cumulative mass losses of all four solidified soils increase with the number of cycles (Figure 4b). The cement- and cement–lime-solidified soils exhibit rapid increases in cumulative mass loss, followed by the cement–sodium silicate-solidified soil, with composite-solidified soil displaying the slowest increase. After ten cycles, the respective total mass losses of the cement-, cement–lime-, and cement–sodium silicate-solidified soils are 4.6, 4.5, and 2.4 g, whereas composite-solidified soil exhibits the lowest total mass loss of only 1.5 g.

Figure 4c shows the variations in the UCSs of the specimens with the number of dry–wet cycles. Under alternating temperature and humidity conditions, the UCSs of all four solidified soil specimens generally decrease as cycling progresses. This is attributed to the damage sustained by the overall structures of the specimens under dry–wet cycling conditions. After ten dry–wet cycles, the respective UCSs of the cement-, cement–lime-, and cement–sodium silicate-solidified soils are 1224, 1142, and 1295 kPa. These values represent the corresponding decreases of 19.5%, 31.9%, and 26.1% in relation to the initial strengths before dry–wet cycling. The UCS of the composite-solidified soil remains relatively high at 2061 kPa after ten dry–wet cycles, marking a decrease of only 6.7% from its initial strength. Upon completion of cycling, the strength of the composite-solidified soil is approximately 1.7 times those of the cement-, cement–lime-, and cement–sodium silicate-solidified soils.

**Figure 5:**
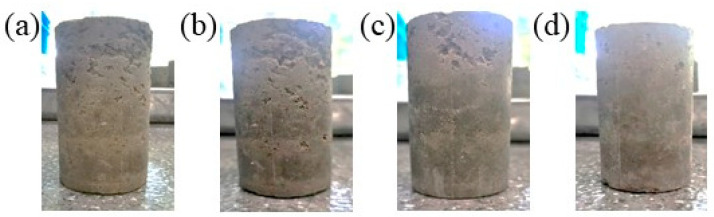
Degrees of damage of the sample surfaces after ten dry–wet cycles: (**a**) 7% cement, (**b**) 7% cement + 0.2% quicklime, (**c**) 7% cement + 0.4% sodium silicate, or (**d**) composite solidifying agent.

During the study, plain soil essentially disintegrates after one dry–wet cycle, rendering the measurement of its strength and mass loss nonviable. Figure 5 shows the levels of surface damage of the cement-, cement–lime-, cement–sodium silicate-, and composite-solidified soils after ten dry–wet cycles. The figure reveals severe surface damage to the cement- and cement–lime-solidified soils under alternating dry–wet cycles. In contrast, the cement–sodium silicate-solidified soil exhibits less damage, and only slight surface damage is observed on the composite-solidified soil. Therefore, the inclusion of modified lignin-based solidifying materials may significantly enhance the durability performance of cement-solidified soil under dry–wet cycling conditions. In comparison, Zhang et al. [31] used 12% lignin to stabilize the soil body, which could resist only four cycles of wetting and drying. This is consistent with the conclusion that lignin should be modified to enhance the durability performance.

### 3.4. Dry Shrinkage Study

**Figure 6:**
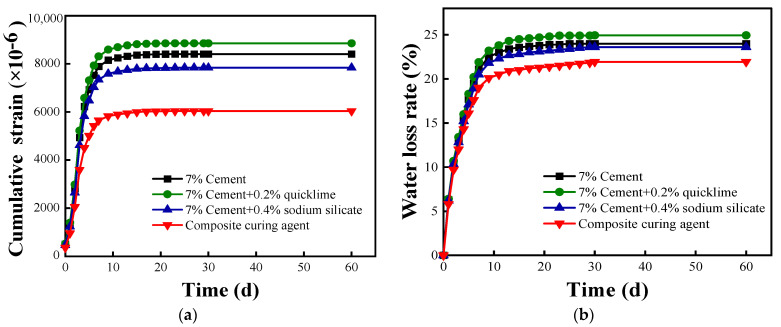
Variations in the shrinkage performance indices with time. (**a**) Shrinkage strain and (**b**) water loss rate.

Figure 6 shows the variations in the dry shrinkage performance indicators of cement-, cement–lime-, cement–sodium silicate-, and composite-solidified soils over time following the addition of the solidifying agents. As shown in Figure 6a, the maximum dry shrinkage strain of cement–lime-solidified soil is marginally larger than that of cement-solidified soil, whereas that of cement–sodium silicate-solidified soil is slightly smaller. A comparison of the dry shrinkage strains of the four solidified soils reveals that composite-solidified soil exhibits a relatively uniform, gentle dry shrinkage strain. Figure 6b shows that the rates of water loss of the four solidified soils increase gradually over time. The final respective rates of water loss of the cement-, cement–lime-, cement–sodium silicate-, and composite-solidified soils are 24.0%, 24.9%, 22.8%, and 21.9%, with composite-solidified soil displaying the lowest rate of water loss.

**Figure 7:**
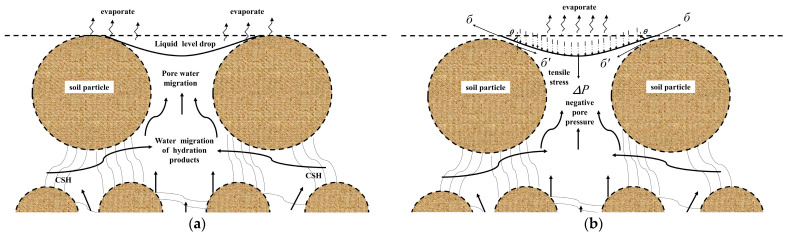
Schematics of the internal changes in the cement-based specimens. (**a**) Schematic of moisture migration in cured soil. (**b**) Schematic of the formation of negative pore pressure.

The dry shrinkage characteristics of a cement-based material are intimately linked to its internal pores. When such a material is relatively dry, the surface moisture begins to evaporate faster than the outward migration of hydration products and water from the internal pores, leading to a decreased level of pore water. As shown in Figure 7a, a curved liquid surface forms between the soil particles, indicating a disruption in the balance of pore water on the surface of the solidified soil. Rapid lowering of the water surface occurs in several tiny capillary pores, resulting in negative pressure and a novel equilibrium point (Figure 7b). Here, the surface tension is essentially the interaction force between a soil particle and the liquid surface. The reaction force, which is equivalent to the negative pore pressure, drives the soil particles inward, causing tensile stress on the pore sidewalls and subsequent pore shrinkage. As evaporation continues, the internal moisture may not compensate for the vacancy of evaporated moisture, again disrupting the balance of the curved liquid surface and causing it to drop further. This decreases the contact angle shown in Figure 7b and enlarges the vertical component of the tensile stress, i.e., the negative pore pressure increases. This cycle repeats, manifesting macroscopically as the overall shrinkage of a dry shrinkage specimen. Over time, the water loss from the specimen extends from the surface to the interior. Cement hydration products, with water absorption capacities similar to those of the soil particles, may not resist the tensile stress caused by the negative pressure, leading to the dry shrinkage of the soil body. The addition of different external materials enables the partial resistance of this tensile stress, thereby reducing the self-shrinkage caused by the drying of cement-based materials [32,33].

Comparisons of the dry shrinkage strain and rate of water loss over time reveal varying effects on the dry shrinkage performance of cement-solidified soil following the addition of different external materials. Notably, the inclusion of quicklime increases the dry shrinkage of cement-solidified soil, whereas it is improved via the addition of sodium silicate. The most significant resistance to tensile stress caused by negative pressure is observed with the addition of the composite solidifying agent, resulting in the smallest dry shrinkage strain in the soil body. Therefore, modified lignin-based materials may also enhance the dry shrinkage performance of cement-solidified soil.

### 3.5. Lightweight DCP Study

**Figure 8:**
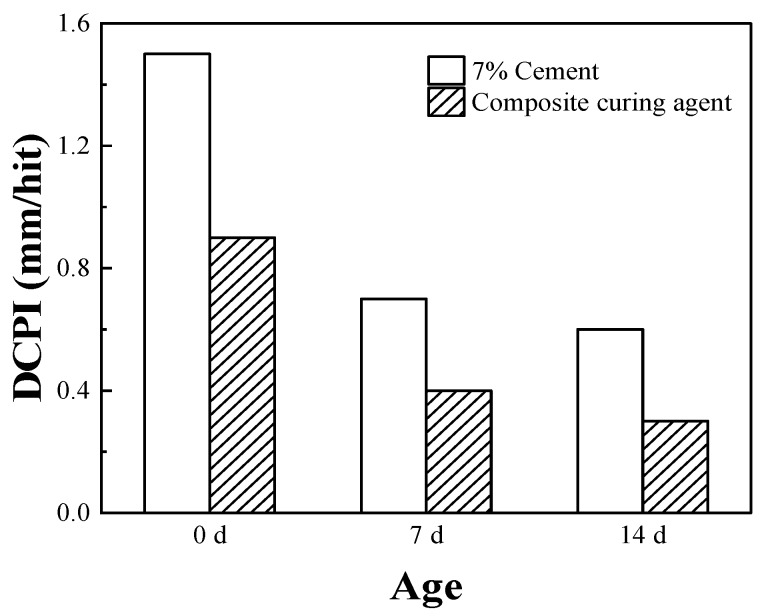
Variations in the dynamic cone penetration indexes with age.

Figure 8 shows the DCPIs of the cement- and composite-solidified soils. At 0 d, the cement- and composite-solidified soils reach their maximum DCPIs, which are 1.5 and 0.9 mm/hit, respectively. As the solidifying age increases, the DCPIs of both soils decrease to varying extents. At 14 d, the DCPI of cement-solidified soil is 0.6 mm/hit, whereas that of composite-solidified soil is only 0.3 mm/hit. This decrease is primarily due to the increased amounts of the hydration products produced by the cement hydration reaction as age increases, which correspondingly enhances the strength of the solidified soil. Concurrently, the external solidifying materials also react, accelerating the increase in the soil strength.

The formulae used in calculating *Rs* are as follows:

(1) $$R_{s}=\frac{W_{s}}{P_{d}}$$

(2) $$W_{s}=\frac{mv^{2}}{2}$$

(3) $$v=\sqrt{v_{0}{}^{2}+2gh}$$

where $W_{s}$ represents the work done by the soil reaction force, which is equivalent to the work done by the falling hammer; $v_{0}$ represents the initial velocity of the falling hammer (0 m/s); *m* represents the mass of the heavy hammer (10 kg); *v* represents the speed of the heavy hammer falling on the anvil (in this study, *v* =$\;\sqrt{2\times 10\times 0.5}=3.16\;$ m/s, *h* = 0.5 m); and *P_d_* represents the distance that the cone head penetrates into the soil.

**Figure 9:**
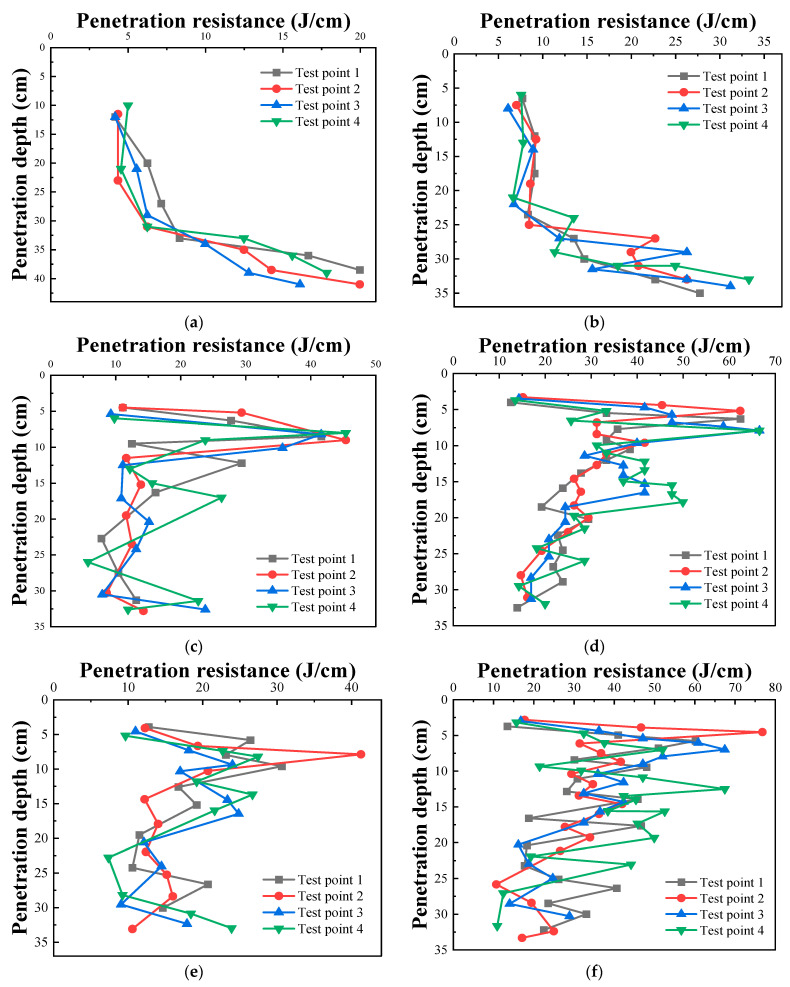
Variations in penetration resistance (*Rs*): (**a**) 0 d hydraulic and (**b**) 0 d composite-consolidated soil *Rs* values, (**c**) 7 d hydraulic and (**d**) 7 d composite-consolidated soil *Rs* values, and (**e**) 14 d soil and (**f**) 14 d composite-consolidated soil *Rs* values.

Figure 9 shows the *Rs* values of the cement- and composite-solidified soils at various ages. A significant increase in resistance is observed when the penetration depth reaches approximately 30 cm, primarily owing to the design thickness of the roadbed soil of approximately 30 cm after rolling and forming at the construction site. When the penetration depth is >30 cm, the probe penetrates into the next soil layer. Notably, the change in *Rs* of solidified soil at 0 d differs from those at 7 and 14 d, where a significant increase in *Rs* is observed when the penetration depth is 5 cm, possibly owing to changes in weather during maintenance. Rainwater seeps into the lower part of the soil layer during rain, whereas the moisture in the upper soil layer evaporates during sunny days. This results in a larger water content in the lower part of the soil layer, causing a smaller resistance in the upper soil layer, a larger resistance in the middle layer, and then a gradual decrease downward. At 0 d, the *Rs* values at four evaluated points in the cement-solidified soil are 10 J/cm, whereas those of the composite-solidified soil are approximately 15 J/cm. At 7 d, the *Rs* values increase significantly to 20 and 33 J/cm for the cement- and composite-solidified soils, respectively. At 14 d, the probe *Rs* values increase further to 24 and 36 J/cm for the cement- and composite-solidified soils, respectively. Therefore, the addition of modified lignin-based external materials significantly enhances the bearing capacity of cement-solidified engineering soil.

### 3.6. Microscopic Analysis

#### 3.6.1. XRD

**Figure 10:**
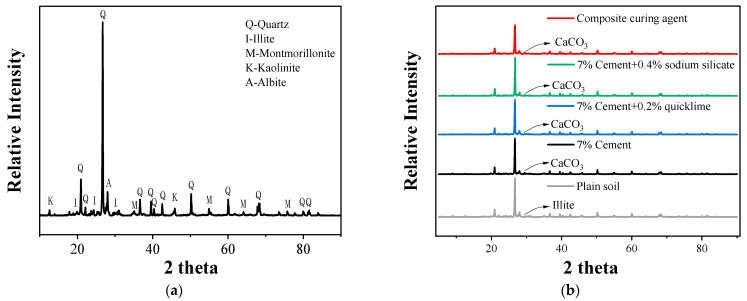
Results of X-ray diffraction analysis of the soils solidified using different materials: (**a**) plain soil and (**b**) plain soil and soils cured with different materials.

Analyzing the XRD patterns of plain and solidified soils after the addition of the four types of improvement agents reveals that the soil sample primarily consists of quartz, illite, montmorillonite, kaolinite, and a small quantity of sodium feldspar. New diffraction peaks at a diffraction angle of 29.5° are observed in the XRD patterns (Figure 10b) of the soil samples after the addition of these agents. This peak may be the characteristic diffraction peak of CaCO_3_, potentially linked to the cement hydration reaction that produces CaCO_3_ and promotes the compact structure of the soil body. The positions of the diffraction peaks in the XRD pattern of the composite-solidified soil are roughly identical to those in the patterns of the cement-, cement–lime-, and cement–sodium silicate-solidified soils, indicating that no new mineral components are produced in the soil body after improvement using the composite solidifying agent. However, decreases in the overall peak intensities of the improved soil are observed, particularly at the diffraction angle 2*θ* = 26.7°, where the intensity of the quartz peak is significantly reduced. This suggests that the sizes of the quartz mineral grains are decreased. These decreases may be associated with the reaction between quicklime and sodium silicate within the composite solidifying agent to create a stronger alkaline environment and partially corrode the quartz. The corrosion products then react with silicate or Ca ions to generate and precipitate amorphous cementitious substances attached to the surfaces of the soil minerals. More cementitious substances are produced in composite-improved soil and attached to the mineral surfaces to fill the pores of the soil body. This explains the enhanced physical and mechanical properties and durability performances of soil bodies treated with composite solidifying agents.

Furthermore, a comparison of the XRD patterns of soils improved with lime, sodium silicate, or cement with that of soil improved with the composite solidifying agent reveals that the diffraction peaks of the latter are the weakest. Therefore, composite solidifying agents display the most significant corrosion–redeposition–cementation effects on soil particles. This may also be related to the increased exposure of the reaction sites on soil minerals under alkaline action and the formation of a more dense and stable structure under the long-chain action of lignin.

#### 3.6.2. SEM

**Figure 11:**
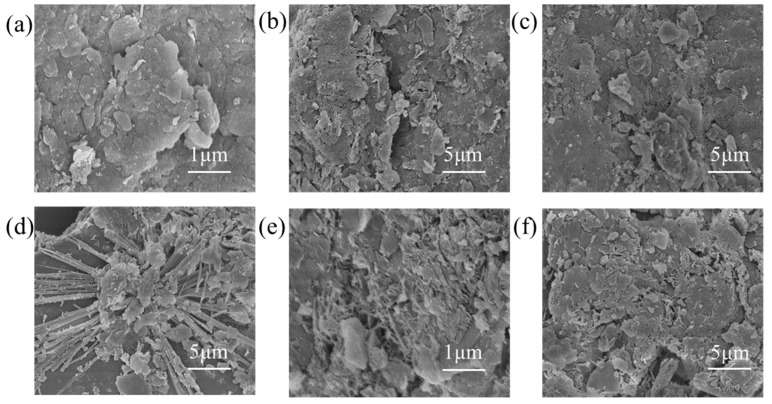
Scanning electron microscopy micrographs of the soils solidified with different materials. (**a**) Plain soil, (**b**) 7% cement, (**c**) 7% cement + 0.2% quicklime, (**d**) 7% cement + 0.4% sodium silicate, and (**e**,**f**) composite solidifying agent.

The SEM images of plain and solidified soils after the addition of various materials reveal that plain soil is interspersed with flaky minerals, such as quartz and montmorillonite (Figure 11). The addition of cement to plain soil results in the appearance of amorphous cementitious substances, which bind the thick, flaky soil structure together. These substances are calcium silicate hydrates (C–S–H) produced by cement hydration, enhancing the bonding forces between the soil bodies and reducing the number of pores. This explains the increase in the strength of the soil body with cement content.

In cement–lime-solidified soil, products similar to those in cement-solidified soil are observed, including amorphous cementitious substances. However, cement–lime-solidified soil contains more compact cementitious substances. In cement–sodium silicate-solidified soil, distinct prismatic crystals are visible. The formation of these hydration products significantly contributes to the cementation of soil particles [34].

Upon the addition of the composite solidifying agent, a thin sheet-shaped cementitious morphology is observed in the soil sample. This morphology, with its compact structure, fills the voids in the soil phase, thereby enhancing the physical and mechanical properties and durability performance of the soil body.

#### 3.6.3. FTIR Spectroscopy

**Figure 12:**
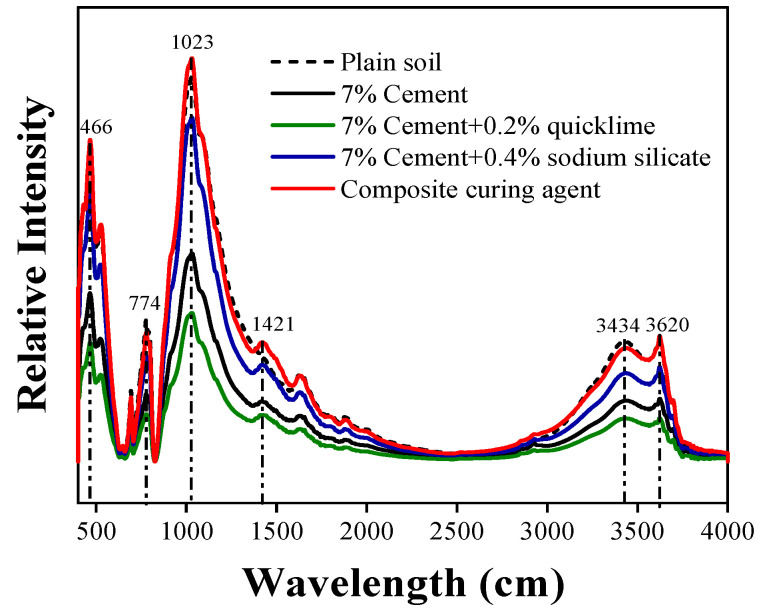
Infrared spectra of the soils solidified using different materials.

The infrared spectra of plain and solidified soil samples after 7 d of solidification with the four types of improvement agents reveal several key observations (Figure 12). In the four improved solidified soils, the wavenumber of the Si–O bond stretching vibration fluctuates: 466, 774, and 1023 cm^−1^. This fluctuation may be attributed to changes in the degree of polymerization of the C–S–H gel generated by the hydration of the cement silicate minerals. The ion exchange between the lignin in the composite solidifying agents and clay minerals may also induce changes in the –OH band (3620 and 3434 cm^−1^) in improved soil [10]. In the range 1350–1500 cm^−1^, peaks at 1421 cm^−1^ are noticeable in the spectra of all four improved solidified soils, but this peak, which represents the C–O vibration, is not observed in the spectrum of plain soil. This is likely due to the carbonation reaction, which also corresponds to the carbonation reaction observed at a diffraction angle of 29.5° in the XRD pattern. Furthermore, compared to those of the other improved soils, no significant changes are observed in the infrared spectrum of solidified soil after improvement using the modified solidifying agent. Therefore, no new functional groups are produced after the addition of composite solidifying agents to cement-solidified soil [35].

In summary, the addition of modified lignin-based external solidifying materials to cement-solidified soil promotes cement hydration and carbonation, thereby enhancing the strength and durability performance of the solidified soil.

The quicklime and sodium silicate in the modified lignin-based external materials generate amorphous calcium silicate, which acts as a nucleus to increase the rate of cement hydration:

(4) $$\mathrm{CaO}+H_{2}O+{\mathrm{Na}}_{2}{\mathrm{SiO}}_{3}\overset{}{\to }{\mathrm{CaSiO}}_{3}↓+2\mathrm{NaOH}$$

NaOH interacts with soil particles to yield sodium silicate, which in turn accelerates cement hydration. Concurrently, more reaction sites on the soil particles are exposed:

(5) $${\mathrm{SiO}}_{2}+2\mathrm{NaOH}\overset{}{\to }{\mathrm{Na}}_{2}{\mathrm{SiO}}_{3}+H_{2}O$$

NaOH reacts with lignin to produce hydroxylated lignin, which wraps and entangles the soil body to compact the slag soil:



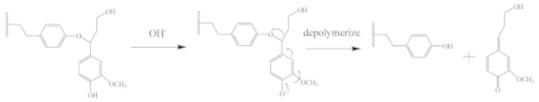

(6)


**Figure 13:**
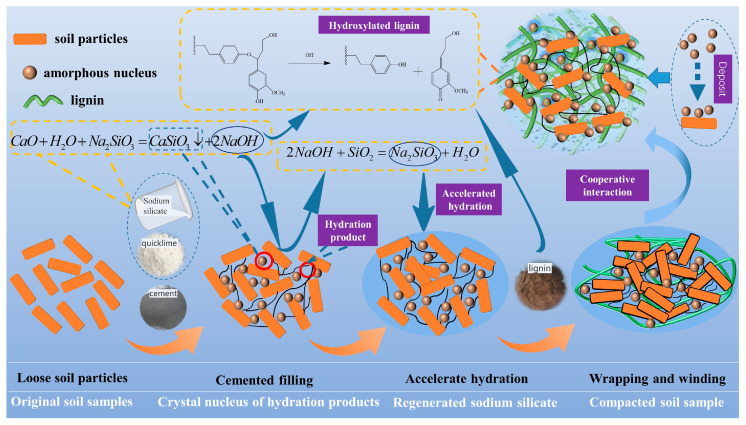
Schematic of the mechanism of action of the solidifying material.

This reaction mechanism is summarized in Figure 13.

### 3.7. Limitations of the Study

Lignin may be modified in various manners, one of which is the use of quicklime and sodium silicate in lignin hydroxylation. The effects of other lignin modification methods, such as etherification and esterification, on the improvement of cement-stabilized soil should be explored. Although the modified lignin-based curing additive used in this study may improve the engineering properties of the engineering soil of the Yangzhou project, several regions exhibit complex engineering geological conditions, and various types of poor soils, such as collapsible loess and saline and expansive soils, are encountered in engineering. Therefore, further research is necessary to determine whether the modified lignin-based composite curing agent may improve the properties of such poor soils. Owing to time constraints, the observation age of the shrinkage study of the modified lignin-based cement-stabilized soil in this work was limited to only 60 d, and further research regarding the long-term strength and durability of the stabilized soil is necessary.

## 4. Conclusions

Lignin, which is a by-product of the paper industry, may moderately enhance the performance of slag soil, even without treatment, but the improvement is limited. This study introduced small quantities of sodium silicate and quicklime as activators to modify lignin and stimulate its activity, thereby yielding solidifying materials for use in enhancing the performance of engineering soil with cement. UCS and direct shear studies revealed that the strength of the soil body may reach 1925 kPa after the addition of the modified lignin-based solidifying material (containing 0.4% sodium silicate, 0.2% quicklime, and 0.2% lignin), which represents 1.5-fold increases in strength and cohesion compared to those of cement-solidified soil, with the internal friction angle increasing by 5.1°. Dry–wet cycling studies indicated that the rate of strength loss of composite-solidified soil was only 35% of that of cement-solidified soil after 10 dry–wet cycles. The maximum dry shrinkage strain (6035 × 10^−6^) of the improved soil after adding the composite solidifying agent was only 70% of the dry shrinkage strain (8405 × 10^−6^) of the cement-solidified soil. The results of the on-site lightweight dynamic penetration studies indicated that the penetration index of composite-solidified soil at 7 d was almost double that of cement-solidified soil. This suggested that the addition of modified lignin-based admixtures could significantly enhance the performance of cement-solidified soil. By analyzing the mechanisms of action of solidifying materials, this study provides a theoretical foundation and technical support for use in applying modified lignin-based materials in the field of solidified soil. Furthermore, this study promotes the application of solidified soil technology in engineering construction.

As most infrastructure should inevitably bear dynamic loads, future research should further explore the dynamic properties of cement-stabilized soil with modified lignin materials to promote the application of modified lignin-based curing additives in foundation engineering. In addition, the influence of cement-solidified soil with modified lignin-based materials on the surrounding environment should be considered.

## Figures and Tables

**Table 1:** Basic physical properties of the soil.

Void Ratio	Natural Water Content (%)	Optimal Water Content (%)	Plastic Limit (%)	Liquid Limit (%)	Plastic Index	Pellets < 0.075 mm (%)
1.25	27.6	15.8	24.64	39.37	14.72	69.39

**Table 2:** Main chemical components of the soil and cement (%).

Sample	CaO	SiO_2_	Al_2_O_3_	Fe_2_O_3_	MgO	SO_3_
Soil	4.88	59.78	18.18	5.45	3.42	0.18
Cement	60.28	21.17	4.26	3.24	3.15	2.74

**Table 3:** Main chemical composition of quicklime.

Composition	CaO	SiO_2_	Al_2_O_3_	Fe_2_O_3_	MgO	Other
Content (%)	81.93	2.97	0.68	0.19	4.72	9.51

**Table 4:** Performance indices of sodium silicate.

Model	SiO_2_ (%)	Na_2_O (%)	Density (g/cm^3^)	Baumé Degree	Modulus	Solid Amount (%)	pH
SP38	26.98	8.53	1.366	38.5	3.3	35.5	10–13

**Table 5:** Schemes for the single-doped cement-based materials.

Curing Agent	Admixture	Dosage (%)	Curing Age (d)
Cement	-	0, 3, 5, 7, 9	7
	Sodium silicate	0.2, 0.4, 0.6, 0.8, 1.0	7
	Quicklime	0.1, 0.2, 0.3, 0.4, 0.5	7
	Lignin	0.1, 0.2, 0.3, 0.4, 0.5	7

**Table 6:** Orthogonal experimental design.

Group	Sodium Silicate (%)	Quicklime (%)	Lignin (%)
1	0.2	0.1	0.1
2	0.2	0.2	0.15
3	0.2	0.3	0.2
4	0.4	0.1	0.15
5	0.4	0.2	0.2
6	0.4	0.3	0.1
7	0.6	0.1	0.2
8	0.6	0.2	0.1
9	0.6	0.3	0.15

**Table 7:** Test scheme mixing scale.

Study	Cement	Cement-Quicklime	Cement-Sodium Silicate	Composite Curing Agent	Age (d)
Direct shear study	7% Cement	7% Cement + 0.2% quicklime	7% Cement + 0.4% sodium silicate	7% Cement + 0.4% sodium silicate + 0.2% quicklime + 0.2% lignin	7
Dry and wet cycling study	7% Cement	7% Cement + 0.2% quicklime	7% Cement + 0.4% sodium silicate	7% Cement + 0.4% sodium silicate + 0.2% quicklime + 0.2% lignin	28
Dry shrinkage study	7% Cement	7% Cement + 0.2% quicklime	7% Cement + 0.4% sodium silicate	7% Cement + 0.4% sodium silicate + 0.2% quicklime + 0.2% lignin	7

**Table 8:** Orthogonal experimental unconfined compressive strength.

Group	1	2	3	4	5	6	7	8	9	Optimal Group
UCS (kPa)	1388	1530	1431	1481	1925	1610	1727	1500	1560	1925

**Table 9:** Soil cohesion (*c*) and internal friction angle (*φ*).

Sample	*c* (kPa)	*φ* (°)
Cement-cured soil	56.2	24.0
Cement–lime-cured soil	62.1	25.0
Cement–sodium silicate-cured soil	67.9	26.2
Composite-cured soil	86.0	29.1

## Data Availability

All relevant data are available within the article and Supplementary Materials.

## Supplementary Materials

The following supporting information can be downloaded at: https://www.mdpi.com/article/10.3390/ma16227100/s1, Figure S1: Influences of the cement content on the unconfined compressive strengths (UCSs) of cement-solidified soil at 7, 14, and 28 d; Figure S2: Influences of the dosage of sodium silicate on the unconfined compressive strengths (UCSs) of cement-solidified soil at 7, 14, and 28 d; Figure S3: Influences of the quicklime content on the unconfined compressive strengths (UCSs) of cement-solidified soil at 7, 14, and 28 d; Figure S4: Influences of the lignin content on the unconfined compressive strengths (UCSs) of cement-solidified soil at 7, 14, and 28 d; Table S1: Results of orthogonal experiments at different ages; Figure S5: Unconfined compressive strengths (UCSs) of the different groups used in the orthogonal study at 7, 14, and 28 d; Figure S6: Relationships between the vertical stresses and shear strengths, as obtained via the 14 and 28 d direct shear studies; Table S2: Levels of cohesion and the internal friction angles of different modifiers at ages of 7, 14, and 28 d; Figure S7: Variations in the dry shrinkage strains of cement-solidified soils under different curing agents in the first 15 d; Figure S8: Variations in the rates of water loss of cement-solidified soils under different curing agents in the first 15 d; Figure S9: Scanning electron microscopy images of plain soil; Figure S10: Analyses of the functional groups of soil and lignin based on Fourier transform infrared spectroscopy.
