# Supplementary material for: Modified Lignin-Based Cement Solidifying Material for Improving Engineering Residual Soil
Source: Materials (Basel). 2023 Nov 9;16(22):7100. doi: 10.3390/ma16227100 (PMC10672641; doi:10.3390/ma16227100)
Supplement: Supplementary file 1 [file materials-16-07100-s001.zip › materials-2673212-supplementary.pdf]

Supporting Information

Modified Lignin-Based Cement Solidifying Material for Improving Engineering Residual Soil

Xiang Yu [1,2], Hongbo Lu [1,*], Jie Peng [2], Jinming Ren [1], Yongmin Wang [1] and Junhao Chen [1]

[1] Power China Huadong Engineering Corporation Limited, Hangzhou 311122, China; yu-xiang@hhu.edu.cn (X.Y.); ren_jm@hdec.com (J.R.); wang_ym2@hdec.com (Y.W.); chen_jh4@hdec.com (J.C.)

[2] College of Civil and Transportation Engineering, Hohai University, Nanjing 210098, China; peng-jie@hhu.edu.cn

* Correspondence: lu_hb2@hdec.com; Tel.: +86-13126859770

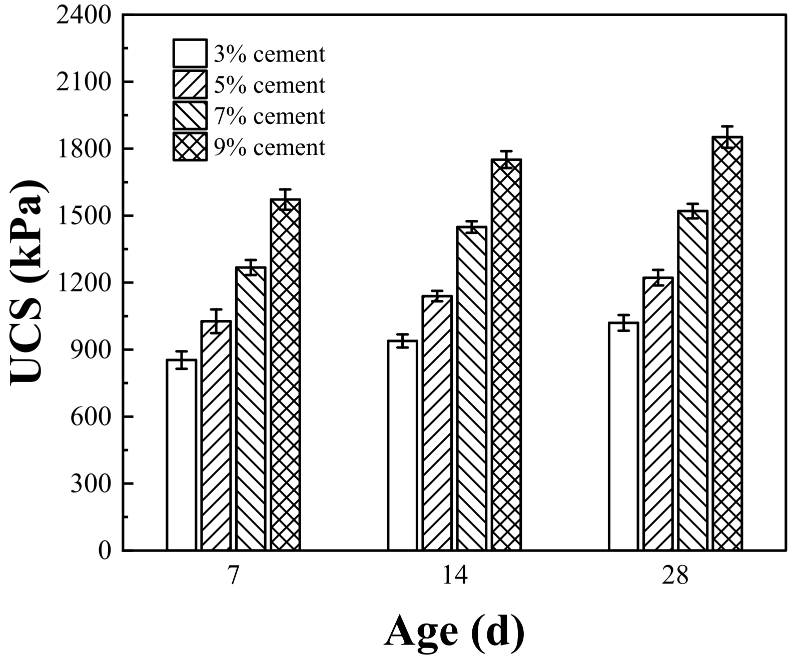

Figure S1. Influences of the cement content on the unconfined compressive strengths (UCSs) of cement-solidified soil at 7, 14, and 28 d.

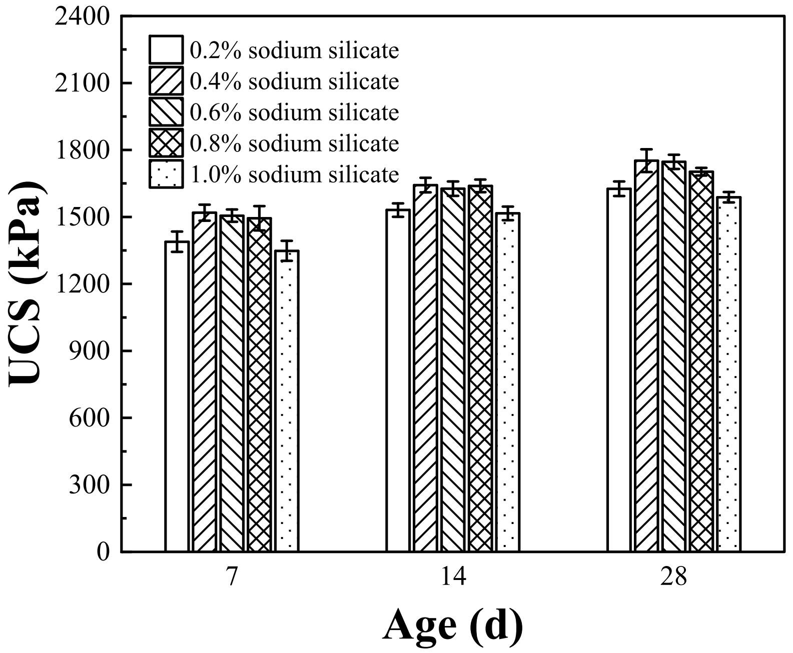

Figure S2. Influences of the dosage of sodium silicate on the UCSs of cement-solidified soil at 7, 14, and 28 d.

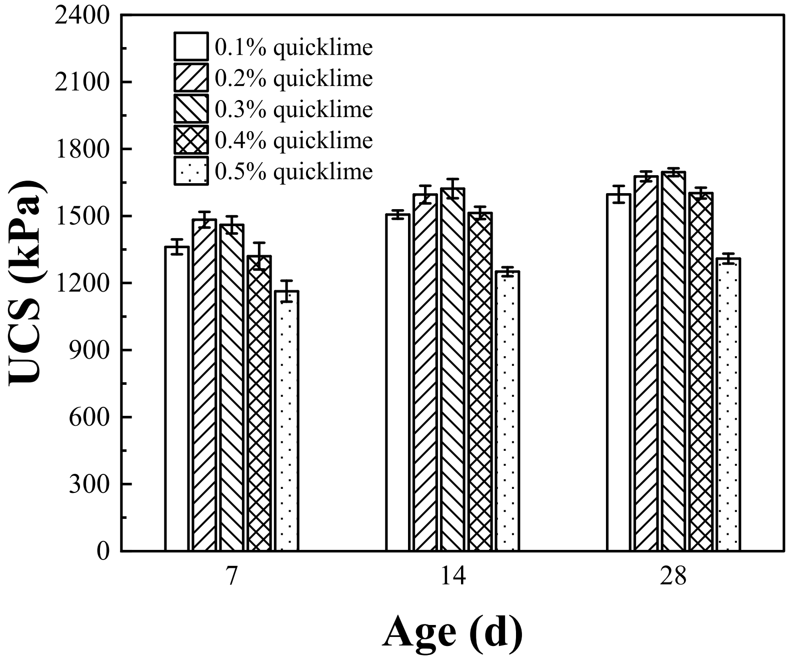

Figure S3. Influences of the quicklime content on the UCSs of cement-solidified soil at 7, 14, and 28 d.

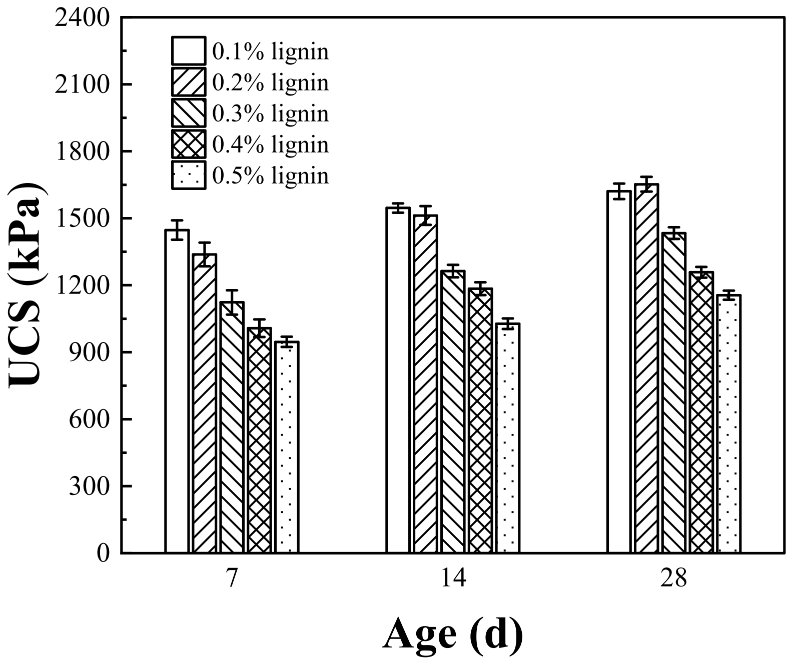

Figure S4. Influences of the lignin content on the UCSs of cement-solidified soil at 7, 14, and 28 d.

Table S1. Results of orthogonal experiments at different ages.

Group	Sodium silicate (%)	Quicklime (%)	Lignin (%)	7 d UCS (kPa)	14 d UCS (kPa)	28 d UCS (kPa)
1	0.2	0.1	0.1	1387	1508	1583
2	0.2	0.2	0.15	1531	1654	1738
3	0.2	0.3	0.2	1432	1670	1746
4	0.4	0.1	0.15	1881	1980	2064
5	0.4	0.2	0.2	1924	2118	2209
6	0.4	0.3	0.1	1611	1834	1918
7	0.6	0.1	0.2	1728	1835	1893
8	0.6	0.2	0.1	1498	1668	1731
9	0.6	0.3	0.15	1561	1664	1820
Optimal group	0.4	0.2	0.2	1924	2118	2209

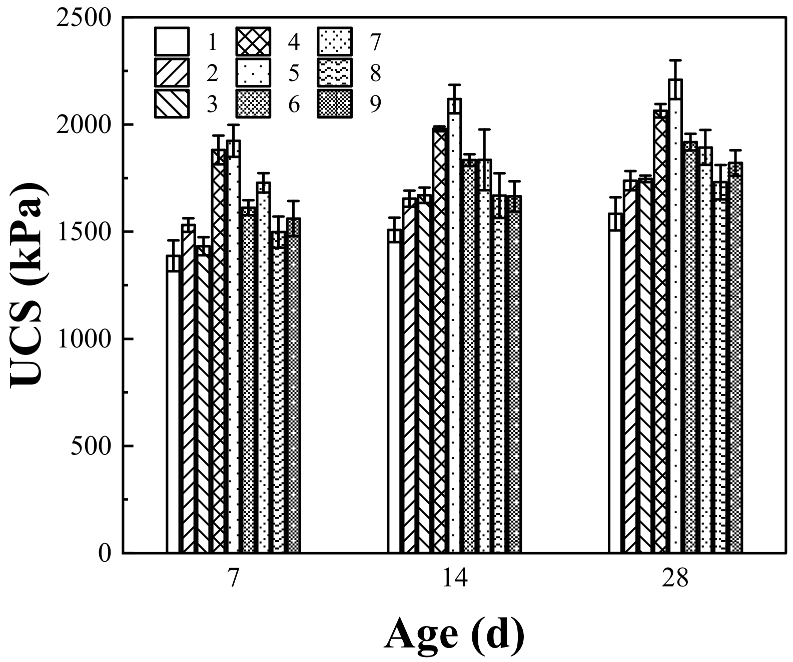

Figure S5. UCSs of the different groups used in the orthogonal study at 7, 14, and 28 d.

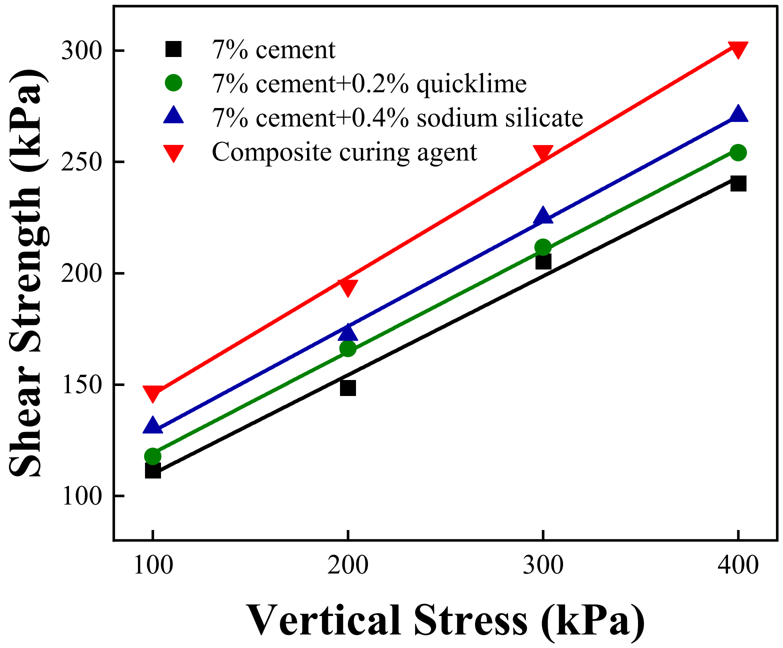

（a）**14 d**

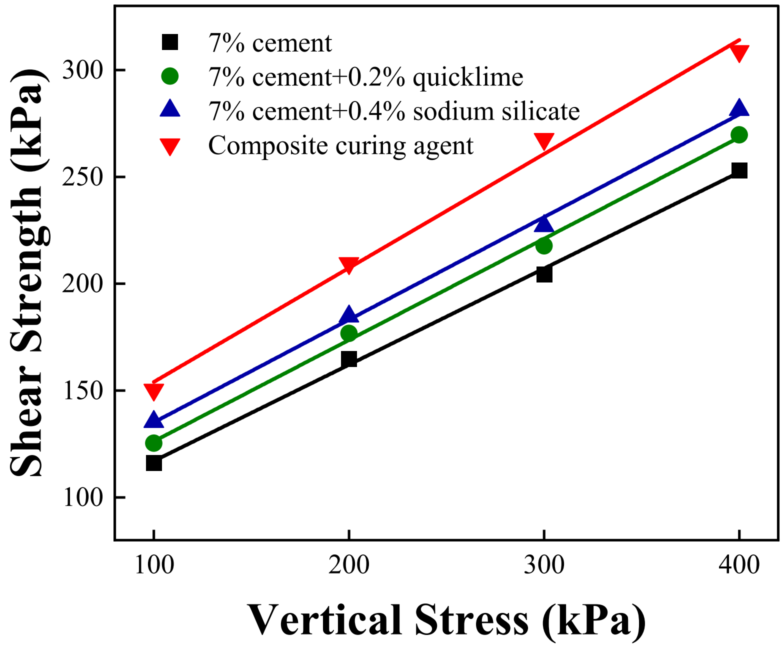

（b）**28 d**

Figure S6. Relationships between the vertical stresses and shear strengths, as obtained via the 14 and 28 d direct shear studies.

Table S2. Levels of cohesion and the internal friction angles of different modifiers at ages of 7, 14, and 28 d.

Curing agent	c （kPa）			φ (°)		
	7 d	14 d	28 d	7 d	14 d	28 d
7 % cement	56.2	67.5	72.0	24.0	25.4	25.8
7 % cement + 0.2 % quicklime	62.1	73.7	78.9	25.0	26.1	27.2
7 % cement + 0.4 % sodium silicate	67.9	81.7	87.1	26.2	27.1	27.5
Composite curing agent	86.0	93.2	100.7	29.1	30.0	30.6

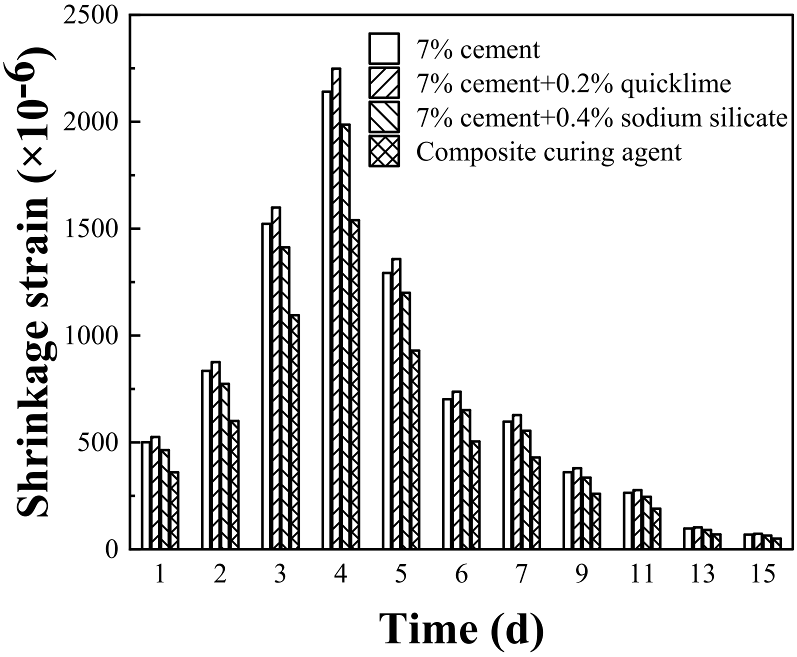

Figure S7. Variations in the dry shrinkage strains of cement-solidified soils under different curing agents in the first 15 d.

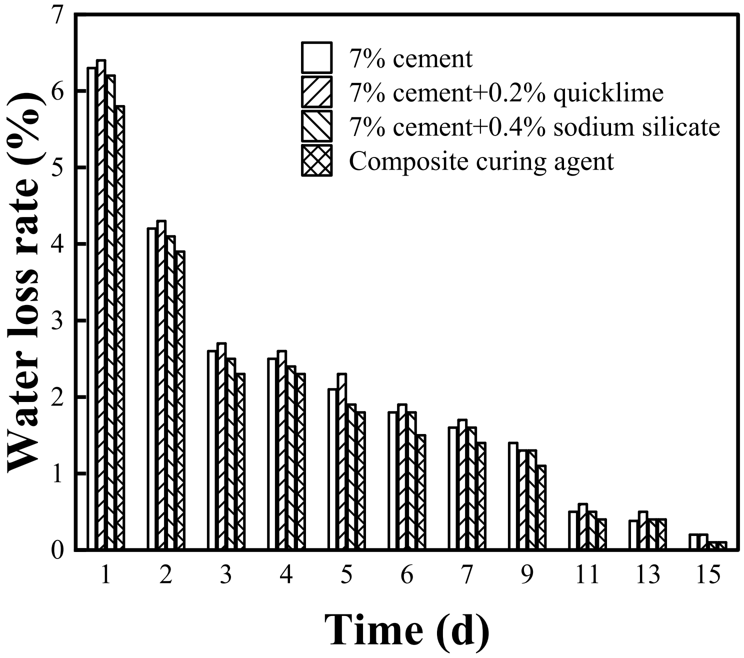

Figure S8. Variations in the rates of water loss of cement-solidified soils under different curing agents in the first 15 d.

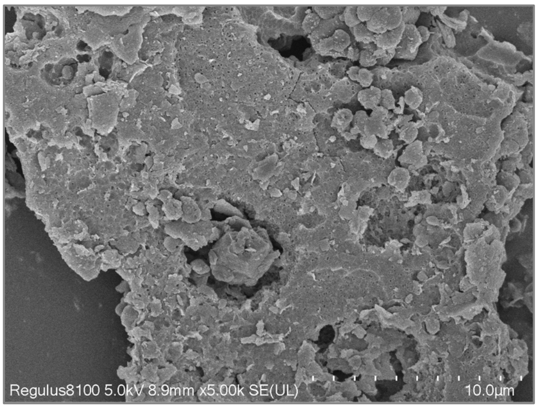

(a) 5000

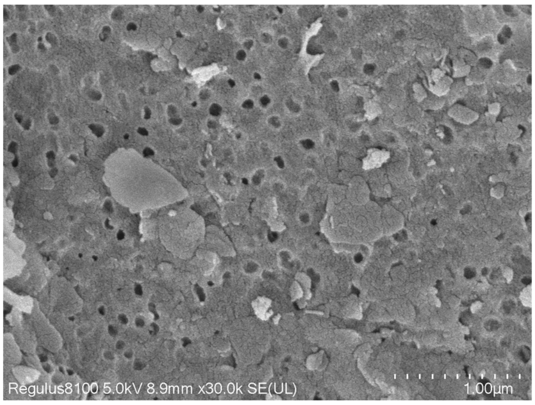

(b) 30000

Figure S9. Scanning electron microscopy images of plain soil at different magnifications.

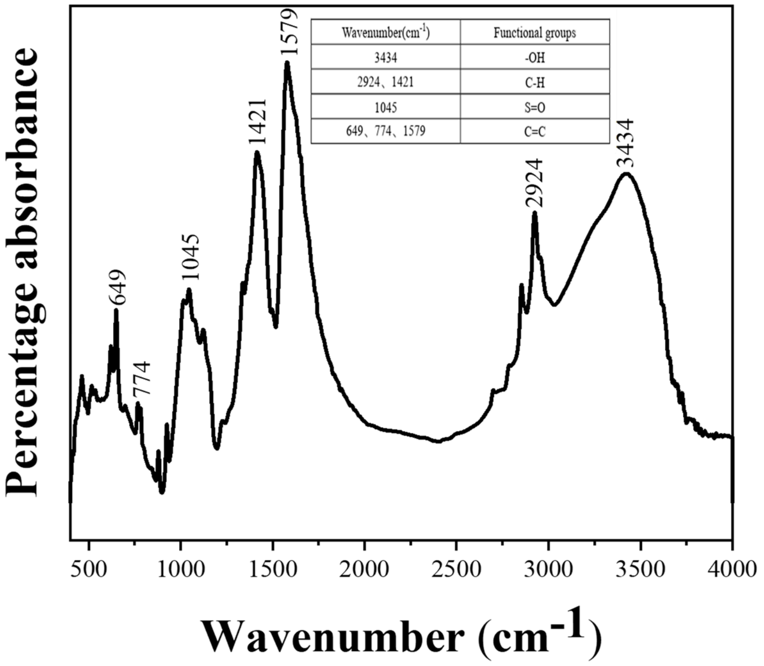

(a) Analysis of lignin functional groups

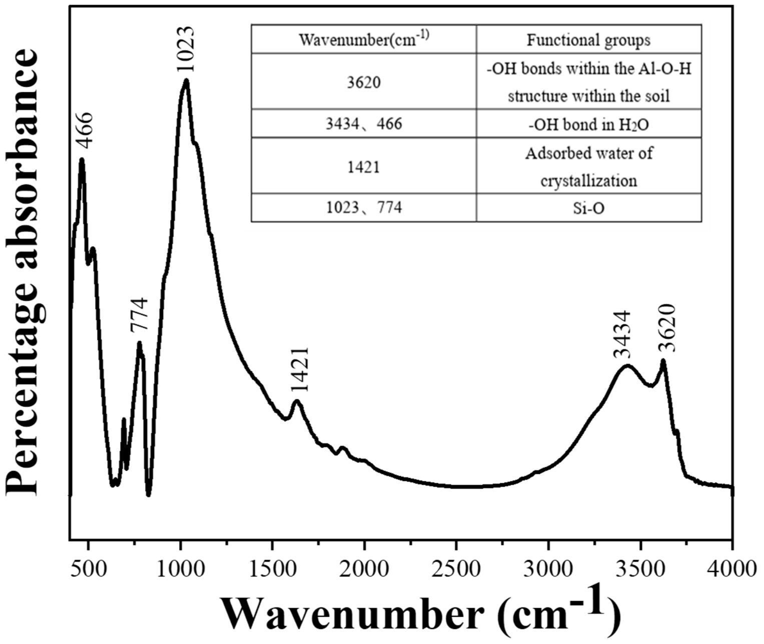

(b) Analysis of soil functional groups

Figure S10. Analyses of the functional groups of soil and lignin based on Fourier transform infrared spectroscopy.
